# Diagnostic Performance of Artificial Intelligence-Based Models for the Detection of Early Esophageal Cancers in Barret’s Esophagus: A Meta-Analysis of Patient-Based Studies

**DOI:** 10.7759/cureus.15447

**Published:** 2021-06-04

**Authors:** Khalid M Bhatti, Zubair S Khanzada, Matta Kuzman, Syed M Ali, Syed Y Iftikhar, Peter Small

**Affiliations:** 1 Surgery, Health Education England, North West, Blackburn, GBR; 2 Surgery, Health Education England, Derby, GBR; 3 Surgery, Health Education England, North East, Newcastle Upon Tyne, GBR; 4 Acute Care Surgery, Hamad General Hospital, Doha, QAT; 5 Surgery, University Hospital of Derby and Burton, Derby, GBR; 6 Surgery, Sunderland Royal Hospital, Sunderland, GBR

**Keywords:** diagnostic performance, artificial intelligence, esophageal cancer, barrett’s esophagus, machine learning

## Abstract

Introduction

Barret’s esophagus (BE) is a precursor of adenocarcinoma of the esophagus. The detection of high-grade dysplasia and adenocarcinoma at an early stage can improve survival but is very challenging. Artificial intelligence (AI)-based models have been claimed to improve diagnostic accuracy. The aim of the current study was to carry out a meta-analysis of papers reporting the results of artificial intelligence-based models used in real-time white light endoscopy of patients with BE to detect early esophageal adenocarcinoma (EEAC).

Methods

This meta-analysis was registered with the International Prospective Register of Systematic Reviews (PROSPERO; Reg No. CRD42021246148) and its conduction and reporting followed the Preferred Reporting Items for Systematic Review and Meta-Analysis of Diagnostic Test Accuracy (PRISMA-DTA) statement guidelines. All peer-reviewed and preprint original articles that reported the sensitivity and specificity of AI-based models on white light endoscopic imaging as an index test against the standard criterion of histologically proven early oesophageal cancer on the background of Barret's esophagus reported as per-patient analysis were considered for inclusion. There was no restriction on type and year of publication, however, articles published in the English language were searched. The search engines used included Medline, PubMed, EMBASE, EMCARE, AMED, BNI, and HMIC. The search strategy included the following keywords for all search engines: ("Esophageal Cancer" OR "Esophageal Neoplasms" OR " Oesophageal Cancer" OR "Oesophageal Neoplasms” OR "Barrett's Esophagus" OR "Barrett's Oesophagus") And ("Artificial Intelligence" OR "Deep Learning" OR "Machine Learning" OR "Convolutional Network"). This search was conducted on November 30, 2020. Duplicate studies were excluded. Studies that reported more than one dataset per patient for the diagnostic accuracy of the AI-based model were included twice. Quantitative and qualitative data, including first author, year of publication, true positives (TP), false negatives (FN), false positives (FP), true negatives (TN), the threshold of the index test, and country where the study was conducted, were extracted using a data extraction sheet. The Quality Appraisal for Diverse Studies 2 (QUADS-2) tool was used to assess the quality of each study. Data were analyzed using MetaDTA, interactive online software for meta-analysis of diagnostic studies. The diagnostic performance of the meta-analysis was assessed by a summary receiver operating characteristics (sROC) plot. A meta-analysis tree was constructed using MetaDTA software to determine the effect of cumulative sensitivity and specificity on surveillance of patients with BE in terms of miss rate and overdiagnosis.

Results

The literature search revealed 171 relevant records. After removing duplicates, 117 records were screened. Full-text articles of 28 studies were assessed for eligibility. Only three studies reporting four datasets met the inclusion criteria. The summary sensitivity and specificity of AI-based models were 0.90 (95% CI, 0.83- 0.944) and 0.86 (95% CI, 0.781-0.91), respectively. The area under the curve for all the available evidence was 0.88.

Conclusion

Collective evidence for the routine usage of AI-based models in the detection of EEAC is encouraging but is limited by the low number of studies. Further prospective studies reporting the patient-based diagnostic accuracy of such models are required.

## Introduction

Esophageal cancer is the 14th most common cancer in the UK, accounting for 3% of all new cancer patients [1]. Between 2015 and 2017, more than 9000 new cases of esophageal cancers were diagnosed [1]. Unfortunately, more than 75% of these patients were diagnosed at stage III or IV; almost 40% of patients had metastatic disease at diagnosis [1]. Late diagnosis is associated with high mortality and reduced one and five-year survival rates i.e., 50% and 20%, respectively [1]. There are two main varieties of esophageal cancers, esophageal adenocarcinoma (EAC) and esophageal squamous cell carcinoma (ESCC), but EAC is more common in the UK and European countries. Risk factors responsible for the development of EAC are numerous, but Barrett’s esophagus (BE) is the most important, as it increases the risk by 30-40-fold [2]. It is hypothesized that due to the reflux of gastric contents into the lower part of the esophagus, the squamous epithelium is replaced by columnar epithelium. Subsequently, over the years, varying degrees of dysplasia develop, which may progress to invasive and metastatic cancers. It is estimated that between 375,000 to 1 million people are suffering from BE [3]. According to Cancer Research UK, 3% to 13 % of patients with BE (8% on average) will develop esophageal adenocarcinoma in their lifetime, giving an annual incidence of around 1% [1].

Early esophageal adenocarcinoma (EEAC) is defined as high-grade dysplasia and adenocarcinoma limited to mucosa only and is of special interest for a variety of reasons. Firstly, the prognosis of EEAC is excellent due to the absence of nodal involvement and metastasis. Secondly, EEAC can be treated using local endoscopic treatments with relatively low complication rates, and most importantly, oesophagectomy can be avoided. However, diagnosing EEAC in the background of Barrett’s esophagus while using conventional white-light endoscopy can be challenging, with a reported miss rate of up to 23%, which leads to delayed diagnosis [4].

There have been various efforts to improve diagnostic accuracy such as the use of narrow-band imaging (NBI), endocytoscopy/ microscopy, endoscopic optical coherence tomography (OCT), and the Seattle protocol for esophageal biopsies [5-6]. Most of these modalities require specialized equipment and their availability is limited. Seattle protocol requires eight random biopsies from every 2 cm of esophagus. This approach is time-intensive and may spoil the field during subsequent endoscopy, making the detection of EEAC even more challenging [7].

For the last decade or so, there has been great interest in developing artificial intelligence (AI)-based models in various fields of medicine including gastroenterology. With reference to endoscopy, AI uses algorithms to analyze endoscopic images and identify validated specific features characteristic of EEAC. Using these features, the program is able to label abnormalities as either benign or malignant. AI may therefore help to increase the diagnostic accuracy of EEAC using widely available white light endoscopy [8-11].

Aims

The aim of the current study is to carry out a meta-analysis of papers reporting the results of AI-based models used in real-time white light endoscopy of patients with BE to detect EEAC.

Objectives

To identify and summarize the evidence on the diagnostic performance of AI-based models using white light endoscopy in the detection of EEAC in patients with BE. These findings can be used to modify and refine the diagnostic capability of AI tissue scanning.

## Materials and methods

For conduction and reporting of the current meta-analysis, Preferred Reporting Items for Systematic Review and Meta-Analysis of Diagnostic Test Accuracy (PRISMA-DTA) statement guidelines were followed [12].

Protocol and registration

This meta-analysis was registered with the International Prospective Register of Systematic Reviews (PROSPERO; Reg No. CRD42021246148).

Eligibility criteria

All peer-reviewed and preprint original articles that reported the sensitivity and specificity of artificial intelligence-based models on white light endoscopic imaging as an index test against the standard criterion of histologically proven early oesophageal cancer in the background of BE reported as per-patient analysis were considered for inclusion. There was no restriction on type and year of publication, however, articles published in the English language were searched.

Information sources

The search engines used included Medline, PubMed, EMBASE, EMCARE, AMED, BNI, and HMIC. For articles where full text was not available, authors were contacted and were reminded after four weeks in case of no reply.

Literature search

Two authors (KB and ZK) independently searched the literature by using the above-mentioned search engines. The search strategy included the following keywords for all search engines: ("Esophageal Cancer" OR "Esophageal Neoplasms" OR " Oesophageal Cancer" OR "Oesophageal Neoplasms” OR "Barrett's Esophagus" OR "Barrett's Oesophagus") And ("Artificial Intelligence" OR "Deep Learning" OR "Machine Learning" OR "Convolutional Network"). This search was conducted on November 30, 2020.

Study selection

Titles and abstracts were independently screened for possible inclusion by the same authors (KB and ZK). Duplicate studies were excluded. Studies that reported more than one dataset per patient for the diagnostic accuracy of the AI-based model were included twice. Studies with the following criteria were excluded: (1) Studies reporting diagnostic accuracy based on images rather than patient analysis; (2) Studies reporting diagnostic accuracy by using techniques other than or in addition to white light endoscopy; (3) Previous meta-analyses, review articles, case reports, letter to editors, abstract-only texts and comments; and (4) Studies where it was not possible to retrieve data clearly reporting the diagnostic accuracy of AI-based models.

The agreement between the two authors was 100%, hence, no statistical methods were used to calculate the level of agreement.

Data collection process

Quantitative and qualitative data, including first author, year of publication, true positives (TP), false negatives (FN), false positives (FP), true negatives (TN), the threshold of the index test, and country where the study was conducted were extracted using a data extraction sheet by both authors (Table 1).

Definitions for data extraction

Adenocarcinoma of the esophagus in patients with Barret's esophagus was considered as the target condition. The reference standard was histologically proven cancer while the artificial intelligence-based diagnosis of cancer was considered as the index test. The cutoff value described by individual studies was taken as the threshold of the index test.

Risk of bias and applicability

The Quads-2 tool was used to assess the quality of each study [13]. The parameters with a risk of bias were patient selection, index test, reference standard, flow and timing; and applicability concerns in terms of the patient selection, index test, and reference standard. In relation to the current meta-analysis, AI-based models used during white light endoscopy were considered index tests while histology-proven EEAC was considered as the reference standard. Patient selection bias was considered to be present if images of high quality were used to assess the accuracy of AI-based models. The index test bias was considered to be present if the threshold was not predefined or if it was adjusted in light of the results of the reference standard.

Diagnostic accuracy measures

Per patient sensitivity and specificity of the AI-based models were used as a measure of diagnostic accuracy.

Synthesis of results

Studies had a minimal variation with reference to the definition of target condition, reference standard used, and threshold of the index test. The reported sensitivity and specificity of each study were combined to perform metaanalysis.

Meta-analysis

Data were analyzed using MetaDTA, interactive online software for meta-analysis of diagnostic studies [14]. The diagnostic performance of the meta-analysis was assessed by a summary receiver operating characteristics (sROC) plot. This is a plot of the true positive rate against the false-positive rate (1-specificity). It also demonstrates mean sensitivity and specificity.

Additional test

A meta-analysis tree was constructed using MetaDTA software to determine the effect of cumulative sensitivity and specificity on surveillance of patients with BE in terms of miss rate and overdiagnosis.

## Results

Study selection

**Figure 1:**
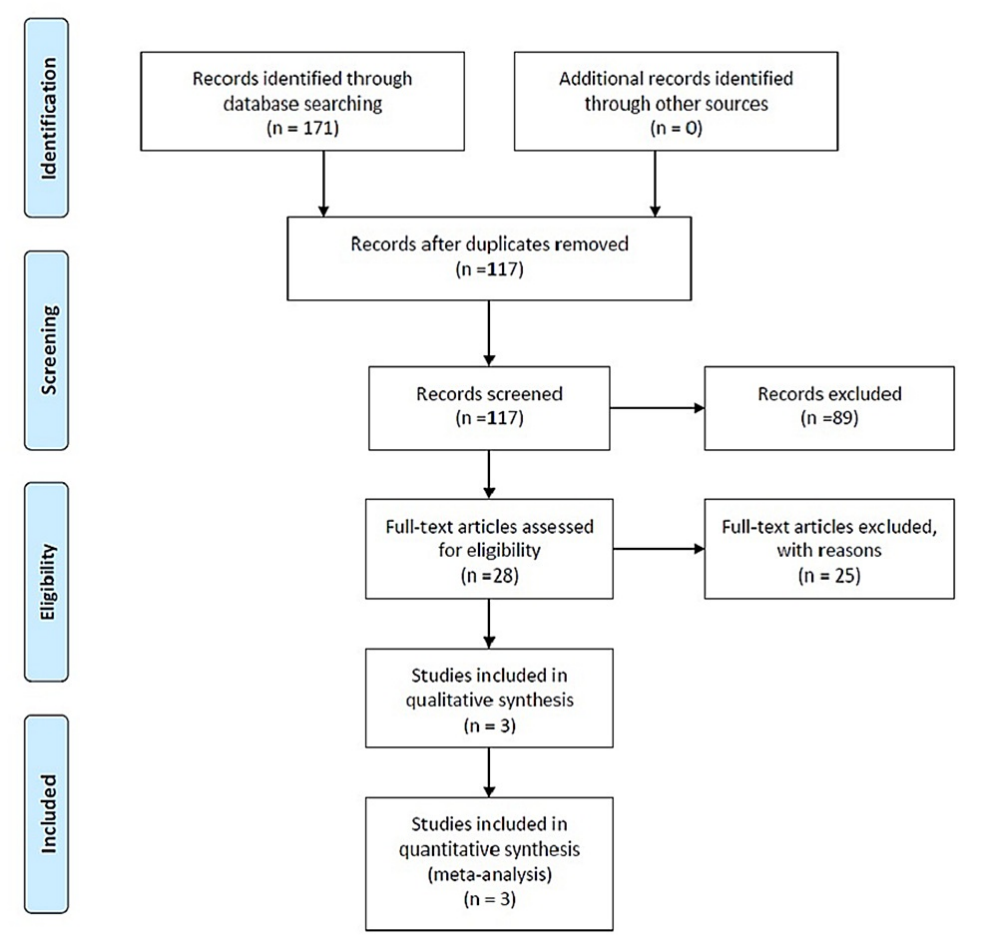
Flow diagram reporting literature and inclusion process

The literature search revealed 171 relevant reports and after removing duplicates, 117 records were screened. Full-text articles of 28 studies were assessed for eligibility. Only three studies reporting four datasets met the inclusion criteria (Figure 1 and Table 1) [15-17]. These studies were also assessed qualitatively using Quads-2.

**Table 1:** Studies included in meta-analysis TP* = True Positive FN** = False Negative FP$ = False Positive TNʃ = True Negative Sens¥ = Sensitivity Spec¥¥ = Specificity

Author	Year	TP*	FN**	FP^$^	TN^ʃ^	Total Number	Sens^¥^	Spec^¥¥^
der Sommen [15]	2016	18	3	3	20	44	0.86	0.87
deGroof-dataset 4 [16]	2020	36	4	5	35	80	0.90	0.88
deGroof-dataset 5 [16]	2020	37	3	7	33	80	0.93	0.83
deGroof [17]	2020	9	1	1	9	20	0.90	0.90

Study characteristics

The first study meeting the criteria of the current meta-analysis was published in 2015 in Endoscopy by Sommen et al. [15]. Using images from 44 patients with Barrett’s esophagus, the AI-based model achieved a sensitivity and specificity of 0.86 and 0.87, respectively. Qualitatively, there was bias at image selection, as images with representative lesions were prominent. Moreover, the threshold for the diagnosis of EEAC was not prespecified and the results of the index test were not interpreted without the knowledge of the results of the reference standard. These flaws in the methodology could have introduced some bias in the performance of the index test.

In 2020, de Groof et al. published a validation study on a similar topic in Gastroenterology [16]. They reported two datasets (datasets 4 and 5) that met inclusion criteria for the current meta-analysis (Table 1). Dataset 4 included 80 patients, and the sensitivity and specificity of the AI-based model were 90% and 88%, respectively. Similarly, dataset 5 included 80 patients and had a sensitivity and specificity of 92.5% and 82.5%, respectively. Qualitatively, the methodology was strong. A threshold was prespecified and results were interpreted by the system without knowledge of the results of the reference standard, i.e. histology. However, the possibility of selection bias could not be ruled out.

The above group (de Groof et al.) published another study in 2020 in Clinical Endoscopy, claiming high accuracy of a previously validated deep learning algorithm in the detection of Barrett’s neoplasia during live endoscopic procedures [17]. The reported sensitivity and specificity were 90%. These results were obtained by assessing the performance of the AI-based model on 20 patients while performing real-time white light endoscopy. Qualitatively, this study was designed and performed to high standards with a predefined threshold and careful patient selection. However, to improve specificity, from 70% to 90%, a majority voting analysis was used instead of a minority analysis.

Pooled sensitivity and specificity

**Figure 2:**
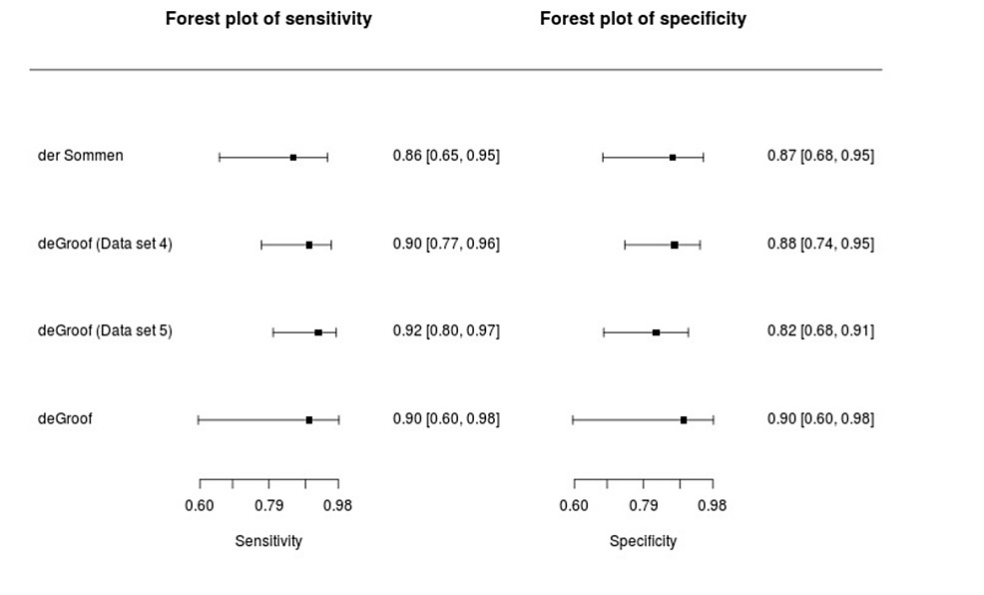
Forest plot for sensitivity and specificity

**Figure 3:**
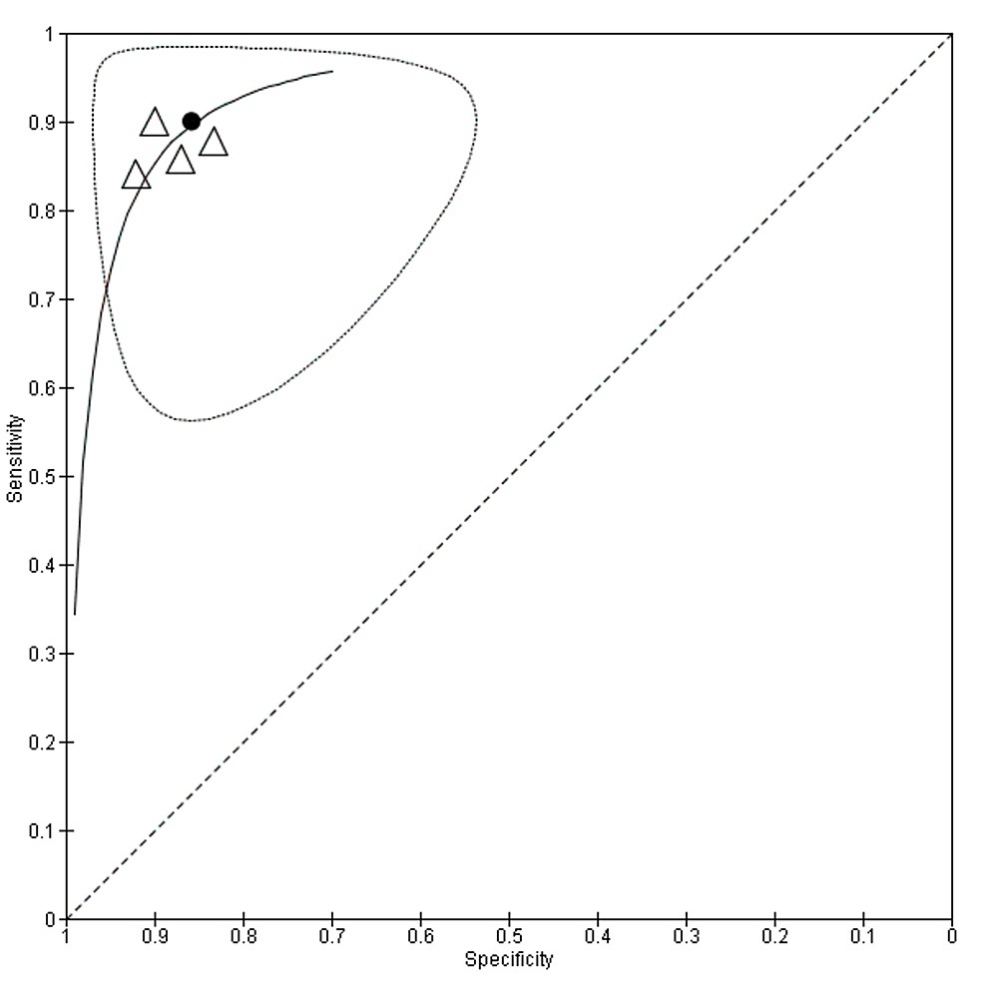
Summary receiver operating characteristics (sROC) plot

The summary sensitivity and specificity of AI-based models in the diagnosis of EEAC in patients with BE were 0.90 (95% CI, 0.83- 0.944) and 0.86 (95% CI, 0.781-0.91), respectively (Figure 2). The summary point with a 95% confidence region and 95% prediction region has been shown in Figure 3. It is evident from the plot that the heterogeneity was minimal. This may be because of the reason that the threshold for three studies was common at 0.6. Figure 3 shows the summary curve for the AI-based models in the detection of EEAC in patients with BE. The area under the curve for all the available evidence was 0.88.

Meta-analysis tree 

**Figure 4:**
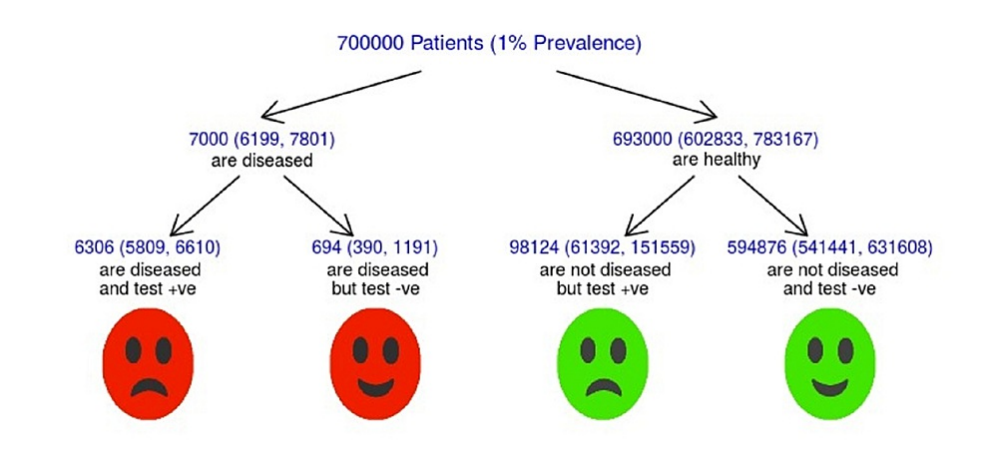
Meta-analysis tree

Figure 4 depicts the meta-analysis tree. Assuming a 1% yearly incidence of esophageal adenocarcinoma in 700,000 (375,000- 1,000,000) patients with BE, it is predicted that 694 patients will be missed and over 98,000 will be overdiagnosed each year by using AI-based models in the detection of EEAC in patients with BE.

## Discussion

To our knowledge, this is the first meta-analysis in the English language assessing the diagnostic performance of AI-based models in the diagnosis of EEAC patients with BE. In this analysis, it was possible to include only four datasets from three studies containing 224 patients due to strict inclusion and exclusion criteria. Only one study, which consisted of 20 patients, was real-time and prospective, whereas the other two were carried out retrospectively [15-17]. A number of studies that reported sensitivity and specificity based on image-based analysis, rather than patient base analysis, were excluded, as the inclusion of such studies would have created selection bias, resulting in clinical applicability issues.

As mentioned earlier, AI-based models not only showed high levels of sensitivity and specificity (0.90 and 0.86) in the diagnosis of EEAC in patients with BE but also a high value of area under the curve (AUC; 0.88). This leads to a summative miss rate of only 10%. Assuming a 1% yearly incidence of oesophageal adenocarcinoma in 700,000 (375,000-1,000,000) patients could result in 694 cancers being missed. This may seem quite high. However, a meta-analysis by Visrodia K et al. reported the magnitude of missed EEAC in patients with non-dysplastic Barret’s using white light endoscopy alone could be as high as 23.9%, meaning that AI-based models are much superior to white light endoscopy alone [4]. A low specificity, on the other hand, could result in a false-positive result in up to 98,000 tests each year using current AI-based models. The workload resulting from this would not be acceptable, mandating an improvement in specificity.

Lui TKL et al. have recently published a meta-analysis of 23 studies reporting the diagnostic accuracy of AI on the detection of gastric and esophageal neoplastic lesions and Helicobacter pylori (HP) status [18]. According to their report, the AUC for BE was 0.96 (95% CI 0.93-0.99) suggesting that the performance of AI was superior to endoscopists. The results were, however, biased by the use of multiple images from few patients. Sensitivity therefore could be skewed by over-diagnosis. In addition, the image review was retrospective and not real-time. Our meta-analysis is based on patient diagnosis, as one would experience in real clinical practice.

Limitations

Our meta-analysis included 224 patients, as only a few studies were available because the use of AI in diagnostic endoscopy is still not widely practiced. Furthermore, we narrowed down the focus of our study specifically on EEAC in BE and interpreted the results accordingly.

Future direction

Our review of current literature has revealed that AI-based image analysis in the diagnosis of EEAC has great potential for widespread clinical use. It can decrease the false-negative rate, shorten diagnostic time, automate endoscopy reporting, and guide biopsy of suspicious areas when compared with simple white-light endoscopy.

However, further work is required to improve automated image analysis and diagnosis of EEAC and reduce the false-positive rate. The following areas should be considered to enable further research and development. 1) Creation of a collaborative network of units interested in developing AI diagnostic software; 2) Establish a large bank of images to train AI software programs; 3) Develop consensus guidelines in order to standardize reporting of AI studies; and 4) Software development, which can be used on standard local computer systems.

The items to be reported and standardized should include the type of scope used, magnification strength of images, annotation software used, pixel quality, number of images per patient, and time duration for automation. Development of these standards would allow for better external validation using prospective double-blinded studies comparing the performance of AI software against more complex modalities currently available.

## Conclusions

This meta-analysis demonstrates that artificial intelligence can improve the early diagnosis of EEAC in Barrett’s esophagus compared with clinician diagnosis using white-light endoscopy alone. Further refinement of software programming is likely to further improve specificity. A sensitivity of over 90% and a negative predictive value of 98% or higher is required to meet regulations suggested by the American Society for Gastrointestinal Endoscopy.

## References

[1] (2020). Cancer Research UK. Esophageal cancer statistics. https://www.cancerresearchuk.org/health-professional/cancer-statistics/statistics-by-cancer-type/oesophageal-cancer.

[2] Schneider JL, Corley DA (2015). A review of the epidemiology of Barrett's oesophagus and oesophageal adenocarcinoma. Best Pract Res Clin Gastroenterol.

[3] (2020). Action Against Heartburn - before it's too late. Persistent heartburn, Barrett's oesophagus and oesophageal cancer. https://www.actionagainstheartburn.org.uk/medical-background/.

[4] Visrodia K, Singh S, Krishnamoorthi R, Ahlquist DA, Wang KK, Iyer PG, Katzka DA (2016). Magnitude of missed esophageal adenocarcinoma after Barrett's esophagus diagnosis: a systematic review and meta-analysis. Gastroenterology.

[5] Sharma P, Hawes RH, Bansal A (2013). Standard endoscopy with random biopsies versus narrow band imaging targeted biopsies in Barrett's oesophagus: a prospective, international, randomised controlled trial. Gut.

[6] Kolb JM, Wani S (2021). Barrett's esophagus: current standards in advanced imaging. Transl Gastroenterol Hepatol.

[7] Elsheaita A, El-Bially MA, Shamseya MM, Ahmed SS, Madkour MA, Shamseya AM, Nouh HH (2020). Seattle protocol vs narrow band imaging guided biopsy in screening of Barrett's esophagus in gastroesophageal reflux disease patients. Medicine (Baltimore).

[8] Lazăr DC, Avram MF, Faur AC, Goldiş A, Romoşan I, Tăban S, Cornianu M (2020). The impact of artificial intelligence in the endoscopic assessment of premalignant and malignant esophageal lesions: present and future. Medicina (Kaunas).

[9] Alagappan M, Brown JRG, Mori Y, Berzin TM (2018). Artificial intelligence in gastrointestinal endoscopy: the future is almost here. World J Gastrointest Endosc.

[10] Zhang YH, Guo LJ, Yuan XL, Hu B (2020). Artificial intelligence-assisted esophageal cancer management: now and future. World J Gastroenterol.

[11] Ruffle JK, Farmer AD, Aziz Q (2019). Artificial intelligence-assisted gastroenterology- promises and pitfalls. Am J Gastroenterol.

[12] Moher D, Liberati A, Tetzlaff J, Altman DG (2009). Preferred reporting items for systematic reviews and meta-analyses: the PRISMA statement. PLoS Med.

[13] (2020). QUADAS-2. https://www.bristol.ac.uk/population-health-sciences/projects/quadas/quadas-2/.

[14] (2020). MetaDTA: diagnostic test accuracy meta-analysis. https://crsu.shinyapps.io/dta_ma/.

[15] van der Sommen F, Zinger S, Curvers WL (2016). Computer-aided detection of early neoplastic lesions in Barrett's esophagus. Endoscopy.

[16] de Groof AJ, Struyvenberg MR, Fockens KN (2020). Deep learning algorithm detection of Barrett's neoplasia with high accuracy during live endoscopic procedures: a pilot study (with video). Gastrointest Endosc.

[17] de Groof AJ, Struyvenberg MR, van der Putten J (2020). Deep-learning system detects neoplasia in patients with Barrett's esophagus with higher accuracy than endoscopists in a multistep training and validation study with benchmarking. Gastroenterology.

[18] Lui TKL, Tsui VWM, Leung WK (2020). Accuracy of artificial intelligence-assisted detection of upper GI lesions: a systematic review and meta-analysis. Gastrointest Endosc.

